# Death by late presenting of diaphragmatic hernia in an infant: case report and review of the literature

**DOI:** 10.1186/s41935-022-00299-x

**Published:** 2022-09-21

**Authors:** Sarra Ben Abderrahim, Maher Jedidi, Amal Ben Daly, Zeineb Nfikha, Mohamed Ben Dhiab, Majed Zemni, Moncef Mokni, Mohamed Kamel Souguir

**Affiliations:** 1 Ibn El Jazzar Faculty of Medicine, The University of Sousse, Mohamed Karoui street, 4002 Sousse, Tunisia; 2 Department of Forensic Medicine, Farhat Hached University Hospital, Ibn El Jazzar street, 4000 Sousse, Tunisia; 3 Department of Pathological Anatomy and Cytology, Farhat Hached University Hospital, Ibn El Jazzar street, 4000 Sousse, Tunisia

**Keywords:** Congenital diaphragmatic hernia, Case report, Infant, Sudden death, Autopsy

## Abstract

**Background:**

Congenital diaphragmatic hernia (CDH) is a congenital malformation of the diaphragm, resulting in the herniation of the abdominal organs into the thoracic cavity. If not properly diagnosed before or at birth, CDH represents a life-threatening pathology in infants and a major cause of death. We present a fatal case of congenital diaphragmatic hernia corresponding to Bochdalek hernia, discovered incidentally during a sudden death’s autopsy of an infant. To achieve a better view of the range of these anomalies, we also conducted a literature review on this subject describing the pathogenesis, manifestations, diagnosis, and autopsy contribution to addressing these CDH.

**Case presentation:**

The case involved a 4-month-old female infant who presented sudden respiratory difficulties after breastfeeding. External examination found marked cyanosis with no evidence of trauma. Upon opening the chest cavity, the stomach, markedly distended, was occupying much of the left pleural cavity. The left lung was compressed and displaced superiorly, and the heart was also compressed and deviated to the right. This mediastinal deviation was due to an ascension of the stomach into the chest cavity through a 2 × 1.5 cm defect in the posterior left hemidiaphragm. Further examination remarked an ecchymotic appearance of the stomach portion entrapped in the hernia defect suggesting recent strangulation of the stomach. The lungs showed atelectasis with signs of pulmonary infection in the histology study.

**Conclusions:**

CDH might be considered uncommon and not always mentioned in the list of sudden death in infant causes. Forensic pathologists should know of this malformation in order to apply the best autopsy techniques and thus allow positive feedback to pediatricians considering the possible legal implications.

## Background

Congenital diaphragmatic hernia (CDH) is an uncommon condition in which the diaphragm does not fully form or fuse during embryologic development, resulting in communication between the thoracic and abdominal cavities (Karamanoukian and Glick 2003; Clugston and Greer 2007; Keijzer and Puri 2010). This anomaly accounts for 8% of all major congenital anomalies, with an incidence of 1 in 2000 to 4000 births (Centers for Disease Control and Prevention 2016) and between 1/2000 and 1/7000 cases in autopsy series (Salaçin et al. 1994; Chhanabhai et al. 1995). The posterolateral Bochdalek hernia is the most common form of CDH (70 to 95%) (Greer 2013) named after Victor Bochdalek, who described patients with this defect in 1848 (Loukas et al. 2008). Although most cases are symptomatic at birth, some cases remain asymptomatic until spontaneous or exacerbated herniation of the abdominal viscera into the thoracic cavity occurs (sometimes until adulthood). A wide spectrum of clinical presentations including various associations of respiratory and gastrointestinal symptoms is described, mainly depending on the nature of the displaced viscera. These late cases (beyond 1 month old) account for 2.6 to 20% of all CDH (Bagłaj and Dorobisz 2005; Muien et al. 2021) and carry a high mortality rate of 20 to 35% (Woodbury et al. 2019; Muien et al. 2021). Due to its milder and more perplexing clinical presentation, this type of CDH poses a significant diagnostic challenge, which can lead to misdiagnosis. In such cases, an autopsy is a valuable means of addressing a misdiagnosis. The undertaken medicolegal investigations enable to draw off an evocative epidemiological profile, through case reports or series of cases. An autopsy is indeed a process that studies both the postmortem dimension and the antemortem history, full of clinical data, sometimes atypical, but which makes all the specificity of this rare case of incidental autopsy findings. We report a fatal case of CDH corresponding to Bochdalek hernia, discovered incidentally during an infant autopsy. In order to achieve a better view of these anomalies, we conducted a literature review on this subject describing the pathogenesis, manifestations, diagnosis, and autopsy contribution to addressing these CDH.

## Case presentation

The case involves a 4-month-old female infant, with no pathological symptoms since birth nor perinatal findings, who presented sudden respiratory difficulties after breastfeeding. The infant reportedly died during transport to pediatric emergencies; thus, no resuscitation maneuvers could be performed at the emergencies arrival. As a result of the unclear cause of death, a medicolegal autopsy was ordered by the prosecutor, and the body was thus transferred for autopsy. A polymerase chain reaction *(*PCR) test for Covid-19 has been performed (postmortem), which was negative.

### Autopsy findings

**Fig. 1:**
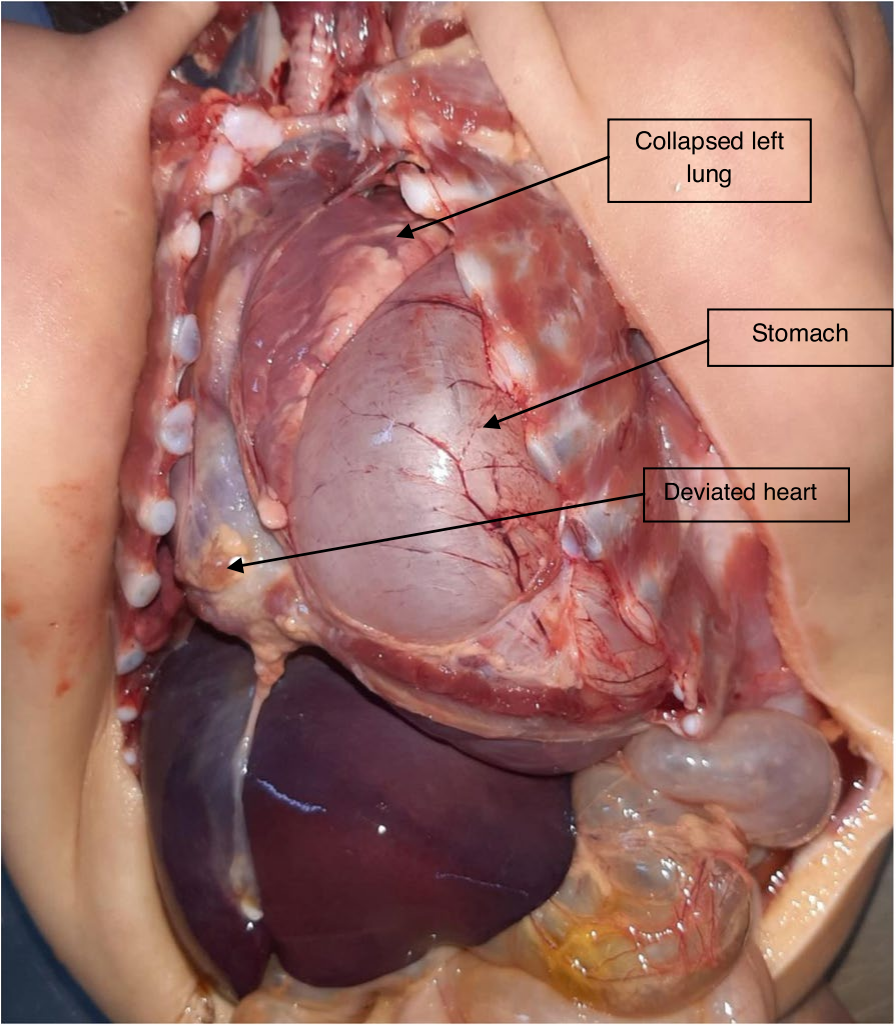
Autopsy findings showing herniated stomach in the chest cavity with severe mediastinal deviation

**Fig. 2:**
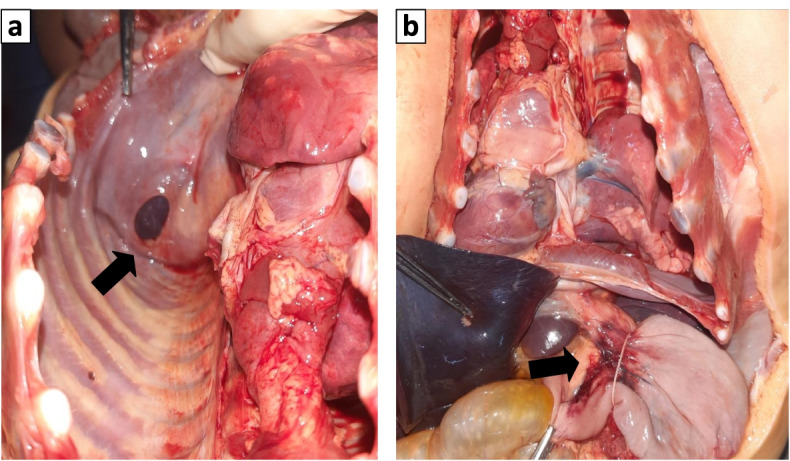
**a** Autopsy findings showing the diaphragm defect (arrow) and ecchymotic stomach portion (lesser curvature) entrapped in the hernia defect (**b**) (arrow)

External examination found marked cyanosis with no evidence of trauma. The body measurements were as follows: bodyweight 7000 g, body length 64 cm, and head circumference 41 cm. Upon opening the chest cavity, the stomach, markedly distended, was occupying much of the left pleural cavity (Fig. 1). The left lung was compressed and displaced superiorly, and the heart was also compressed and deviated to the right. This mediastinal deviation was due to an ascension of the stomach into the chest cavity through a 2 × 1.5 cm defect in the posterior left hemidiaphragm (Fig. 2a). A white creamy material was found (150 mL) within the stomach, supporting the history of breastfeeding when the episode occurred. The right hemidiaphragm was intact with no evidence of laceration or hemorrhage of the diaphragm, and no pleural or pericardial effusions were present. Further examination remarked an ecchymotic appearance of the stomach portion entrapped in the hernia defect (Fig. 2b) suggesting recent strangulation of the stomach. The left lung weighed 30 g, and the right lung weighed 50 g. No heart (weighing 35 g) defect was found at dissection nor any other defect on the remaining autopsy findings.

### Postmortem investigations

**Fig. 3:**
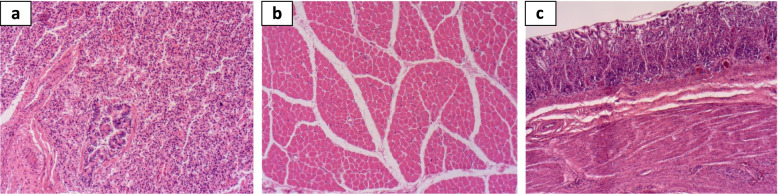
Histology findings of different organs, hematoxylin and eosin (HE) coloration. **a** Alveolar collapse with polymorphic inflammatory infiltrate and vascular congestion (HE × 100). **b** Normal diaphragm muscle cells component (HE × 100). **c** Chronic inflammatory changes of the gastric mucosa with vascular congestion (HE × 100)

The histology study showed alveolar collapse with the presence of a polymorphic inflammatory infiltrate of the left lung (Fig. 3a) consistent with atelectasis associated with signs of pulmonary infection. The diaphragm histology study (Fig. 3b) showed normal muscle cells component, while chronic inflammatory changes of the gastric mucosa with vascular congestion were found (Fig. 3c). No toxic substances were detected in the toxicology study, ruling out the possibility of poisoning which could explain the death.

The cause of death was concluded as a consequence of acute respiratory failure related to a diaphragmatic hernia with severe mediastinal deviation.

## Discussion

The reported case describes the death of an infant due to late-presenting CDH which was incidentally discovered during the autopsy. Delayed presentation in late childhood or adolescence represents a dilemma in diagnosis, as 16% of infants with late-presenting CDH are reported to have previously normal chest radiography, and initial radiographic findings are misinterpreted in 25 to 62% of cases (Kadian et al. 2009; Kumbhar et al. 2019). As such, it may be an overlooked cause of mortality. This was not our case, as the infant did not report any respiratory symptoms since birth according to her parents.

The pathogenesis of CDH is not yet fully understood (Klaassens et al. 2009; Kosiński and Wielgoś 2017); however, diaphragm defects are suspected to result from aberrations in the development of the pleuroperitoneal folds (PPF) and pericardial-peritoneal ducts, an anomaly in the proliferation of the mesenchymal components of the PPF, or impaired migration of somite-derived pre-muscular and nerve cells (Kosiński and Wielgoś 2017). The defect occurs very early in pregnancy (10–12 weeks of gestation), which corresponds to the time of the diaphragm development (Sefton et al. 2018) and is detected through a routine ultrasound at 22 to 28 weeks of gestation. The diaphragmatic structures do not extend toward each other or fuse during development, resulting in an incomplete diaphragm or a complete diaphragm with insufficiently muscled and therefore weak regions (Solomon and Hayes 2016). In our case and according to the histology study, the diaphragm has normally developed with normal muscular cells, except for the defect area which corresponded to an incomplete diaphragm structure.

The most common form of CDH is the Bochdalek hernia (85% of all types of CDH) (Tartar et al. 2018) as supported by the literature review (Table 1). This anomaly accounts for 8% of all major congenital anomalies, with an incidence of 1 in 2000 to 4000 births (Centers for Disease Control and Prevention 2016), the majority being approximately 3 cm in diameter (Lally et al. 2007; Tartar et al. 2018). They occur posteriorly and are due to a defect in the posterior attachment of the diaphragm when there is a failure of pleuroperitoneal membrane closure in utero. They are frequently left sided (Tartar et al. 2018), owing to a later diaphragm closure in fetal life than the right side, which may also explain the asymmetric occurrence. Other types of CDH include the Morgagni hernia (~27%) and central hernia (~2–3%) (Mehollin-Ray 2020) (Table 1). Morgagni hernias refer to the herniation through the foramen of Morgagni (i.e., small defects in the posterior aspect of the anterior thoracic wall between the sternal and costal attachments of the diaphragm). When compared to Bochdalek hernias, Morgagni hernias tend to be anterior, more often right sided (~90%), and at low risk of prolapse (Mohamed et al. 2020). In our case, the defect corresponded to a Bochdalek hernia, and a missing hernia sack reported at autopsy suggested a congenital defect. Usually, large Bochdalek’s hernias are associated with pulmonary hypoplasia resulting in respiratory distress, while small Bochdalek’s hernias may allow normal lung development and thus remain asymptomatic until the occurrence of a triggering event (De et al. 2013). In this case, it is presumed that the hernia was initially small at birth, causing few or no symptoms. The precipitating event that ultimately led to acute respiratory decompensation was an airway infection in the poorly ventilated atelectatic areas of the collapsed lung. When the stomach reached its full capacity (after feeding), it exerted a mechanical compression on the ipsilateral lung causing severe mediastinal deviation and compression of the heart and upper respiratory tracts. The important volume of the herniated abdominal content caused an obstructive shock and asphyxia, consistent with the marked cyanosis found at the body examination.

**Table 1:** Literature review of neonatal and pediatric CDH

Cases/authors	Age	Gender	Hernia site	Symptoms
Series of cases (*n* = 26)/Woodbury et al. (Woodbury et al. 2019)	Mean age: 35.9 ± 6.5 weeks of gestation	F (*n* = 16) M (*n* = 10)	Right (*n* = 5) Left (*n* = 17) Bilateral (*n* = 2)	-
Case report/Hmadouch et al. (Hmadouch and Barkat 2020)	Day 4 of life	F	Left (Bochdalek hernia)	Breathing difficulties at 2 days old
Autopsy study (*n* = 13)/Borys et al. (Borys and Taxy 2004)	Min: 10 days old Max: 31 weeks of gestation	F (*n* = 9) M (*n* = 4)	Left (*n* = 11) Right (*n* = 2)	-
Case report (autopsy case)/Mobilia et al. (Mobilia et al. 2013)	3 years old	M	Left (Bochdalek hernia)	Breathing difficulties, hypertonia, trismus, subsequent loss of consciousness
Case report (autopsy case)/Solomon et al. (Solomon and Hayes 2016)	6 weeks old	F	Left (Bochdalek hernia)	Breathing difficulties after breastfeeding
Series of cases (*n* = 79)/Doyle et al. (Doyle and Lally 2004)	38 ± 2.8 weeks of gestation	F (*n* = 27) M (*n* = 50)	Right (*n* = 21) Left (*n* = 53)	Respiratory symptoms (*n* = 20) Gastrointstatinal symptoms (*n* = 15) Both (*n* = 6) Asymptomatic (*n* = 5)
Case report (autopsy case)/Chau et al. (Chau et al. 2013)	3 months old	F	Left (Bochdalek hernia)	Tachypnea, fatigue
Series of cases (*n* = 7)/Clugston et al. (Clugston and Greer 2007)	Mean age: 48 months old	F (*n* = 3) M (*n* = 4)	Left (*n* = 7)	Gastrointestinal symptoms (*n* = 6) Respiratory symptoms (*n* = 1)
Case report (autopsy case)/Bolino et al. (Bolino et al. 2015)	40 weeks of gestation	M	Left (Bochdalek hernia)	Died after delivery (absence of spontaneous breathing at birth)
Case report/Blibech et al. (Blibech et al. 2014)	Day 1 of life	M	Right	Neonatal respiratory distress
Case report/Uinarni et al. (Uinarni et al. 2020)	Born at term	F	Right	Cyanosis, breathing difficulties after birth
Case report/Lava et al. (Lava et al. 2012)	Born at term	F	Left (Bochdalek hernia)	Sudden respiratory distress after birth
Case report (autopsy case)/Kotis et al. (Kotis et al. 2009)	5.5 months old	F	Left	Fever, tachypnea, tachycardia
Case report/Ghabisha et al. (Ghabisha et al. 2021)	6 months old	M	Left	Low-grade fever, poor feeding, intermittent respiratory distress, cough, vomiting, nausea
Case report/Xia et al. (Xia et al. 2017)	34 weeks of gestation	F	Left	Respiratory distress
Case report/Kalvandi et al. (Kalvandi et al. 2018)	3 years old	M	Left	Epigastric pain after a meal
Series of cases (*n*=4)/Ananda et al. (Kesavan et al. 2017)	Min: 15 months old Max: 4 years old	F (*n* = 2) M (*n* = 2)	Left (Bochdalek hernia) (*n* = 2) Right (Morgani hernia) (*n* = 1) Hiatus hernia	Recurrent respiratory tract infection, fever and cough, breathlessness, retrosternal chest pain
Case report (*n* = 2)/Lemos et al. (Lemos et al. 2015)	11 months old And 4 months old	F (*n* = 1) M (*n* = 1)	Left (Bochdalek hernia) Right hernia	Distended abdomen, fever, dyspnea, cyanosis

*F* Female, *M* Male

From a forensic point of view, herniation of abdominal contents into the pleural cavity may be a postmortem artifact caused by pressure buildup in the abdominal cavity due to putrefaction (with a compromise of the structural integrity of the diaphragm), by cardiorespiratory resuscitation maneuvers before death, by direct penetrating injury (gunshot/stab injuries), or less commonly, secondary to blunt abdominal trauma (motor vehicle accidents, falls, crush injuries) (James et al. 1999; Thompson et al. 2016; Elibol et al. 2022). Forensics should hence be aware that some hernias are acquired, and any traumatic origin must first be eliminated before considering the hypothesis of a congenital hernia. Ultimately, a herniation may be unrelated to death. In our case, there was no traumatic context nor any performed resuscitation maneuvers reported before death. There were also no abdominal nor thoracic organ injuries at autopsy findings suggesting blunt trauma. CDH was retained as the cause of death due to lung compression with respiratory failure and/or mediastinal shift after postmortem investigations.

The autopsy here was crucial for elucidating the cause of death. As such, the autopsy is a means of providing an accurate understanding of the cause of death to minimize outcome bias. It provides families and medical caregivers with objective explanations for some respiratory distress etiologies that might be considered uncommon and not always mentioned in the main clinical guidelines (Chau et al. 2013). As late-presenting diaphragmatic hernia is associated with a wide range of clinical symptoms (Table 1), some manifestations such as difficulty breathing, cyanosis (blue color of the skin), abnormal chest development (with one side being larger than the other), or abdomen that appears caved in should make clinicians suspect this anomaly. This is all the more pertinent before developmental delays such as delays in the ability to roll over, sit, crawl, stand, or walk.

## Conclusions

In conclusion, the presented case corresponds to a late-presenting CDH incidentally discovered during a sudden death autopsy. Symptoms revealing this defect were respiratory distress caused by the herniation of all the stomach into the chest cavity. Forensic pathologists should know of this malformation in order to apply the best autopsy techniques and thus allow positive feedback to pediatricians considering the possible legal implications. CDH will be retained as the cause of death only after eliminating all other etiologies of acquired herniation of the abdominal organ into the chest cavity.

## Data Availability

Not applicable

## Abbreviations

CDH: Congenital diaphragmatic hernia
COVID-19: Coronavirus disease 2019
PCR: Polymerase chain reaction
